# Whole-Genome Sequence of a Suid Herpesvirus-1 Strain Isolated from the Brain of a Hunting Dog in Italy

**DOI:** 10.1128/genomeA.01333-16

**Published:** 2016-12-01

**Authors:** Federica Pizzurro, Iolanda Mangone, Guendalina Zaccaria, Eliana De Luca, Daniela Malatesta, Marco Innocenti, Irene Carmine, Francesca Cito, Maurilia Marcacci, Daria Di Sabatino, Alessio Lorusso

**Affiliations:** a Dipartimento di Scienze Veterinaria, Università di Pisa, Teramo, Italy; b Istituto Zooprofilattico Sperimentale dell’Abruzzo e Molise (IZSAM), Teramo, Italy; c Veterinary Practitioner, L’Aquila, Italy

## Abstract

Suid herpesvirus-1 (SHV-1), a DNA virus of the family *Herpesviridae*, causes a severe and fatal disease in a wide range of mammals. Here, we report the whole-genome sequence of an SHV-1 isolated in Italy in 2014 from the brain of a hunting dog that suffered from an acute and severe disease.

## GENOME ANNOUNCEMENT

Aujeszky’s disease (AD) is an infectious disease sustained by suid herpesvirus-1 (SHV-1) which belongs to the family *Herpesviridae*, genus *Varicellovirus*. The genome consists of a single linear molecule of double-stranded DNA (1).

The disease affects many animal species causing acute encephalitis that evolves into the death of the infected individuals. SHV-1 is associated primarily with pigs, the natural host, which remain latently infected following clinical recovery except piglets under two weeks old, which die from encephalitis. AD is endemic in many parts of the world, but several countries have successfully completed eradication programs in pigs, e.g., United States, Canada, New Zealand, and many member states of the EU. The disease is controlled by containment of infected herds and by the use of vaccines or removal of latently infected animals (2). However, SHV-1 is being continuously reported in the wild boar population across Europe and in hunting dogs (3).

On November 24, 2014, a seven-year-old Segugio Maremmano dog, used for hunting in the province of L'Aquila, Abruzzi region, Central Italy (N 42.36769 E 13.24154), came in contact with the blood of a wild boar. The next day, the dog showed intense pruritus concentrated in the region of the face mainly around ears and muzzle. Death occurred after a fatal and acute disease on November 26 and the carcass was sent to the IZSAM for diagnosis.

DNA purified from brain homogenate tested positive for SHV-1 by real time PCR (ADIAVET PRV REALTIME kit); the homogenate was subsequently employed for viral isolation on RK13 cells. Strain ADV32751/Italy2014 was successfully isolated at second cell passage and confirmed by real time PCR and immunofluorescence. Then, genomic DNA was purified (Qiagen DNeasy Blood & Tissue Kit) and quantified with the Qubit DNA HS assay kit (Thermo, Fisher Scientific). One nanogram was used for library preparation by using the Nextera XT library prep kit (Illumina Inc.) according to the manufacturer’s protocol. Deep sequencing was performed on the NextSeq 500 (Illumina, Inc.) using the NextSeq 500/550 Mid Output Reagent Cartridge v2, 300 cycles and standard 150 bp paired-end reads.

A total of 3,272,891 paired-end reads were obtained. Reads were trimmed and assembled using a combined approach. *De novo* assembly was conducted by SPAdes v3.1.0 followed by mapping against a reference SHV-1 strain (NC_006151) using Mira v4.0.2. The mean coverage of the obtained consensus sequence representing the whole genome of ADV32751/Italy2014 (142,276 bp) was 841×. Previous studies demonstrated that the Italian SHV-1 strains are divided into three clusters (4) and that clear genetic distinction between strains isolated from hunting dogs exposed to wild boars and those originated from working dogs, with domestic pigs as likely sources, does exist (5). By phylogenetic analysis of partial gC gene sequences, ADV32751/Italy2014 apparently belongs to the Italian cluster 1 with other SHV-1 strains isolated from wild boars and hunting dogs in Italy (4). To date, as far as we know, this is the ﬁrst whole-genome sequence of an SHV-1 strain isolated in Italy. It will certainly support additional work on the diagnosis, evolution, and epidemiology of SHV-1.

### Accession number(s).

The nucleotide sequence for ADV32751/Italy2014 has been deposited in GenBank under accession number KU198433.
